# Anterior Skull Base Tuberculosis Mimicking an Anterior Skull Base Meningioma: A Case Report

**DOI:** 10.7759/cureus.75218

**Published:** 2024-12-06

**Authors:** Saif A Badran, Aous Mohammad Qasim, Mohammed Thakir Ismail, Ali Shahadha, Ahmed A Al-Juboori

**Affiliations:** 1 Department of Surgery, Ibn Sina University of Medical and Pharmaceutical Sciences, Baghdad, IRQ; 2 Department of Surgery, College of Medicine, Ninevah University, Mosul, IRQ; 3 Department of Neurosurgery, Dr. Sa’ad AL-Witri Hospital for Neurosciences, Baghdad, IRQ

**Keywords:** anterior skull base, central nervous system tuberculosis, granulomatous inflammation, meningioma mimicry, neuroimaging

## Abstract

Tuberculosis (TB) affecting the central nervous system (CNS) is rare, often mimicking other intracranial pathologies such as meningiomas, especially when located in the anterior skull base. Despite a global reduction in TB incidence, CNS TB continues to present diagnostic challenges due to its nonspecific imaging characteristics. We report a case of a 39-year-old male with symptoms including persistent headache, vertigo, and visual disturbances. Initial imaging suggested meningioma; however, histopathology revealed a tuberculoma. The patient responded well to antitubercular therapy following surgery.

This case emphasizes the necessity of considering TB in differential diagnoses of intracranial lesions, especially in endemic regions, to prevent misdiagnosis and ensure timely treatment. CNS TB, though rare, should be a differential consideration in intracranial lesions to avoid delays in diagnosis and improve clinical outcomes.

## Introduction

Tuberculosis (TB) is a leading cause of infectious disease morbidity and mortality worldwide, especially in low- and middle-income countries. Although pulmonary TB is the most common presentation, extrapulmonary manifestations are increasingly recognized, comprising about 15-20% of TB cases in immunocompetent individuals and a higher percentage in immunocompromised populations [1]. Within these extrapulmonary cases, central nervous system (CNS) TB represents a rare but severe form of the disease, occurring in roughly 2-5% of all TB cases and accounting for high morbidity due to its complex presentation and delayed diagnosis [1].

CNS TB typically presents as tuberculous meningitis, intracranial tuberculomas, or spinal TB, each with distinct clinical and radiologic characteristics. Tuberculous meningitis, the most common form of CNS TB, involves inflammation of the meninges and is associated with severe neurological deficits [2]. Tuberculomas, on the other hand, are granulomatous lesions that can mimic other space-occupying intracranial lesions, such as neoplasms, often resulting in diagnostic challenges. Tuberculomas may present with symptoms such as headache, vomiting, and altered mental status, making them difficult to distinguish from other intracranial pathologies [2].

Recent data suggest that advancements in diagnostic imaging, such as MRI and CT, have improved the detection of TB-related CNS involvement. However, differentiating CNS TB from neoplastic conditions like meningiomas remains challenging, especially in cases affecting the anterior skull base [3]. Meningiomas are the most common primary intracranial tumors and can present with a clinical profile similar to tuberculomas, leading to potential misdiagnosis. Misinterpretation of TB lesions as neoplastic can delay appropriate treatment, increasing the risk of neurological complications and mortality [3].

The epidemiology of TB has seen a significant shift globally over the past decade. In countries like Iraq, there has been a notable decline in TB incidence due to effective public health interventions, yet cases of CNS TB persist [4]. These cases underscore the importance of considering TB in the differential diagnosis of atypical brain lesions, even in populations with declining TB incidence. The present case highlights the rare presentation of TB in the anterior skull base, initially suspected to be a meningioma, underscoring the diagnostic complexity and necessity for a high index of suspicion in similar cases.

## Case presentation

A 39-year-old male patient with a history of hypertension presented with a two-month history of progressively worsening headaches, accompanied by vertigo, dizziness, blurred vision, and tinnitus. His symptoms were not associated with abnormal body movements. Given the presence of these neurological symptoms, an initial diagnosis of meningioma was considered, and he was admitted for surgical intervention.

**Figure 1:**
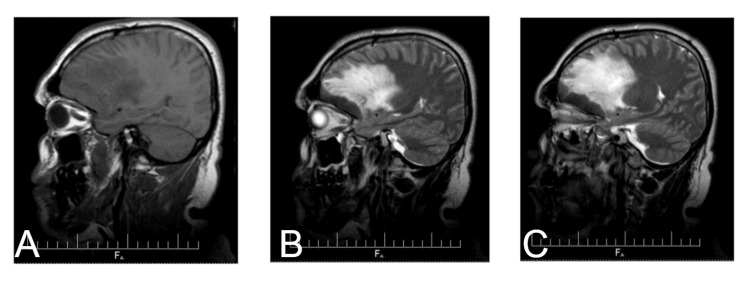
Sagittal MRI images of the brain. (A) An MRI T1-weighted image sagittal view shows a hypo- to iso-intense lesion associated with edema. The lesion appears well-defined, without irregular borders, which is consistent with meningiomas. (B) A T2-weighted image shows lesion at the anterior skull base, involving the frontal region. (C) Further delineation of the lesion shows increased hyperintensity (brain edema) in the T2 and involvement of the sphenoid wings and optic chiasm.

**Figure 2:**
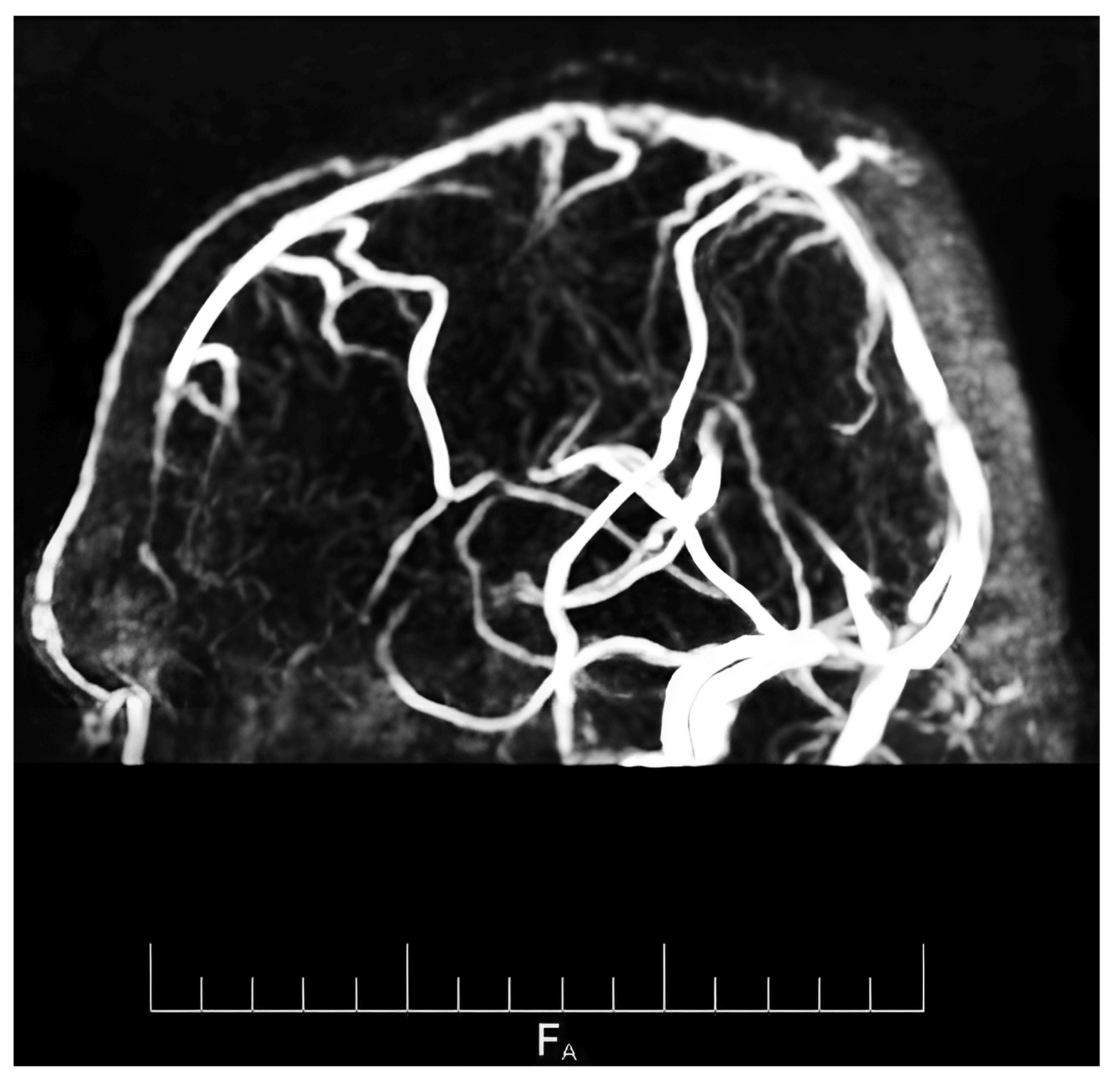
MRV of the brain demonstrates that the vascular pattern is preserved, showing normal branching and flow signal intensity without irregularities or disruptions. MRV: Magnetic resonance venography

**Figure 3:**
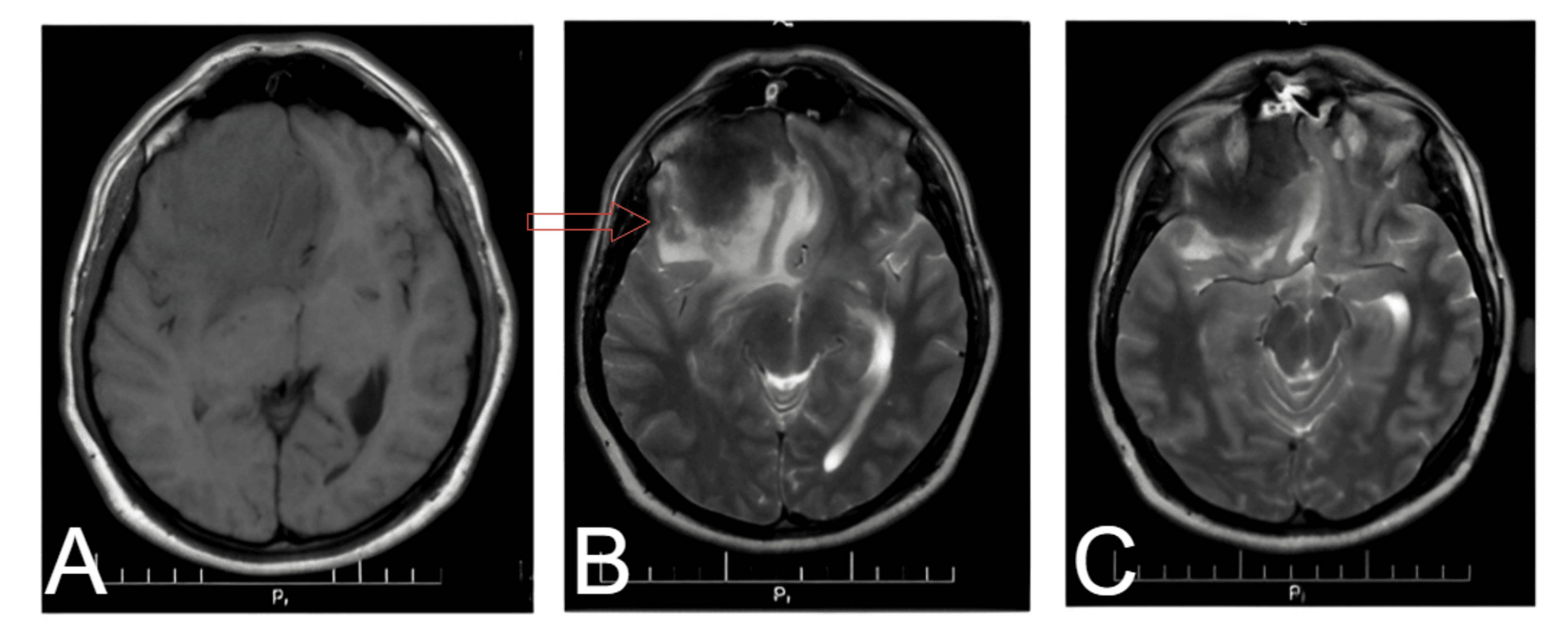
Axial MRI images demonstrate a frontal skull base lesion with associated T2 hyperintensity and perilesional edema, suggestive of a granulomatous pathology. (A) There is a lesion in the frontal skull base. (B) The lesion is hyperintense on T2-weighted imaging, indicating edema or increased water content in the surrounding brain parenchyma. (C) The lesion's involvement of adjacent structures, including the skull base and potential compression of the optic nerves, is more clearly seen.

Upon examination, imaging studies, including an MRI with gadolinium enhancement, revealed a mass lesion located at the frontal skull base. A chest X-ray revealed no findings (Figures 1-3).

The lesion extended from the crista galli back to the optic foramina and sphenoid wings, particularly affecting the right side and causing bilateral papilledema due to optic nerve compression.

The anterior skull base lesion was well-demarcated on the MRI. The mass demonstrated marked enhancement on imaging with gadolinium enhancement, indicative of a highly vascularized or inflammatory lesion. The lesion was well-circumscribed, with sharp demarcation from surrounding brain tissue, often seen in neoplastic or granulomatous pathologies.

T2-weighted imaging showed hyperintensity, indicative of associated vasogenic edema or increased water content within the lesion and adjacent brain parenchyma. Surrounding perilesional edema extended into nearby structures and may have contributed to the patient's neurological symptoms, such as headache and visual disturbances.

On axial imaging, the lesion was visualized to involve the frontal skull base with posterior as well as lateral extensions toward both sphenoid wings down to the optic foramina; this location and involvement may well explain the bilateral papilledema, very possibly due to compression of the optic nerves and subsequent increased intracranial pressure.

Magnetic resonance venography (MRV) of the brain was performed, which showed no vascular irregularities or flow disruption, hence excluding vascular anomalies/involvement within the lesion. These findings reduced the likelihood of differential diagnoses such as arteriovenous malformations or cavernomas.

The patient underwent surgery under general anesthesia using a right-sided lateral supra-orbital approach with craniotomy. During the procedure, a total microresection of the lesion was achieved, with coagulation of the frontal skull base dura. Intraoperatively, the lesion was suspected to be a tuberculoma rather than a meningioma, prompting further histopathological analysis. During the surgical resection, the lesion exhibited a friable consistency with areas of caseation, which is atypical for the fibrous and firm nature of meningiomas. The histopathology confirmed the presence of a tuberculoma with granulomatous inflammation and caseating necrosis, consistent with TB. Section showed confluent epithelioid caseating granuloma with multiple Langhans multinucleated giant cells and necrosis with fibrosis.

Upon review of the patient's medical records and history, there was no documented evidence of prior TB.

Postoperatively, the patient’s recovery was uneventful. He had no cerebrospinal fluid (CSF) leakage, and there were no complaints of headache, weakness, or personality changes. However, anosmia was noted as a residual symptom. On discharge, he was prescribed a regimen including Keppra (500 mg twice daily), Decadron (1 ampoule daily), Doliprane (1000 mg twice daily), omeprazole (20 mg daily), and ceftriaxone (1000 mg daily).

We did not start immediately with the TB therapy while awaiting the histopathological confirmation to ensure diagnostic accuracy. The patient was clinically stable, and there were no systemic symptoms of TB that would suggest a need for empirical therapy. An internal medicine consultation was obtained, and the prescribed medications were given after confirmation of diagnosis.

## Discussion

TB of the CNS is a rare manifestation, occurring in only 2-5% of TB cases. Tuberculomas, which are even less common than tuberculous meningitis, pose significant diagnostic challenges due to their ability to mimic neoplastic lesions such as meningiomas. CNS TB cases tend to be associated with high morbidity, particularly in cases where diagnosis is delayed, as seen with lesions in atypical locations like the anterior skull base [1]. In this case, the presentation initially suggested meningioma based on MRI findings, underscoring the diagnostic complexity and the necessity of considering TB as a differential diagnosis, especially in endemic regions [2].

The imaging characteristics of tuberculomas and meningiomas can overlap considerably. MRI findings in this patient showed features suggestive of meningiomas. Among the key features of meningiomas, the lesion was extra-axial. Other features suspicious for meningioma included contrast enhancement, well-circumscribed margins, and mass effect, although the lack of a dural tail sign and other signs made the diagnosis less clear. Symptoms, including headache, blurred vision, and vertigo, were related to raised intracranial pressure, which is reported in approximately 47% of CNS TB cases. In addition, bilateral papilledema in this case further indicated intracranial involvement, aligning with reported symptoms of CNS TB [3]. Histopathology, revealing granulomatous inflammation with caseating necrosis, was crucial in establishing a definitive diagnosis, highlighting the importance of biopsy in cases with ambiguous imaging results [4].

The imaging and clinical presentation of anterior skull base TB often overlaps with those of meningiomas, particularly in their dural attachment and mass effect nature. This diagnostic challenge underscores the need for a multidisciplinary approach, combining advanced imaging and histopathological confirmation. Recent studies have highlighted the variability in meningioma symptomatology, which depends on factors such as tumor grade, size, and location, further complicating the differential diagnosis of intracranial lesions. These findings support the consideration of uncommon causes, like TB, in cases with imaging features mimicking neoplastic pathologies [5].

Global TB incidence has decreased significantly over the past decade due to advancements in TB control. In Iraq, for example, TB incidence fell from 45 cases per 100,000 people in 2013 to 23 cases per 100,000 in 2023. Despite this, extrapulmonary TB, including CNS involvement, remains a challenge. Extrapulmonary TB cases account for about 15-20% of all TB presentations worldwide, with CNS TB presenting one of the highest risks for morbidity [6].

The present case emphasizes the need for clinicians to maintain a high index of suspicion for TB in patients with atypical intracranial extra-axial lesions, even in regions where TB prevalence is declining. Prompt diagnosis and initiation of antitubercular therapy are essential as treatment delays can lead to severe neurological complications. This case underscores the critical role of a multidisciplinary approach, involving imaging, histopathology, and clinical correlation, to achieve accurate diagnosis and improve patient outcomes [7].

## Conclusions

This case highlights the importance of including TB in the differential diagnosis of intracranial lesions, particularly in rare locations like the anterior skull base. Although TB incidence has declined in many regions, cases of central nervous system TB still pose significant diagnostic challenges due to their potential to mimic neoplastic conditions, such as meningiomas. Early diagnosis and initiation of antitubercular therapy remain essential to improving outcomes and minimizing complications. Clinicians should maintain vigilance for TB in atypical presentations to ensure accurate diagnosis and prompt management.
